# Unsupervised Facial Action Representation Learning by Temporal Prediction

**DOI:** 10.3389/fnbot.2022.851847

**Published:** 2022-03-16

**Authors:** Chongwen Wang, Zicheng Wang

**Affiliations:** School of Computer Science, Beijing Institute of Technology, Beijing, China

**Keywords:** facial action unit recognition, self-supervised learning, contrastive learning, temporal predictive coding, representation learning

## Abstract

Due to the cumbersome and expensive data collection process, facial action unit (AU) datasets are generally much smaller in scale than those in other computer vision fields, resulting in overfitting AU detection models trained on insufficient AU images. Despite the recent progress in AU detection, deployment of these models has been impeded due to their limited generalization to unseen subjects and facial poses. In this paper, we propose to learn the discriminative facial AU representation in a self-supervised manner. Considering that facial AUs show temporal consistency and evolution in consecutive facial frames, we develop a self-supervised pseudo signal based on temporally predictive coding (TPC) to capture the temporal characteristics. To further learn the per-frame discriminativeness between the sibling facial frames, we incorporate the frame-wisely temporal contrastive learning into the self-supervised paradigm naturally. The proposed TPC can be trained without AU annotations, which facilitates us using a large number of unlabeled facial videos to learn the AU representations that are robust to undesired nuisances such as facial identities, poses. Contrary to previous AU detection works, our method does not require manually selecting key facial regions or explicitly modeling the AU relations manually. Experimental results show that TPC improves the AU detection precision on several popular AU benchmark datasets compared with other self-supervised AU detection methods.

## 1. Introduction

Facial expression recognition technology offers the opportunity to seamlessly capture the expressed emotional experience of humans and facilitates unique human-computer interaction experiences. Over the past decades, facial expression recognition and analysis have been a hot research topic in the field of computer vision and human-computer interaction. To precisely characterize facial expressions, Ekman *et al*. developed the facial action coding system (FACS) (Ekman and Friesen, 1978). FACS has been widely used for describing and measuring facial behavior and has been the most comprehensive, anatomical system for describing facial expressions. FACS defines a detailed set of about 30 atomic non-overlapping facial muscle actions, i.e., action units (AUs). Almost any anatomical facial muscle activity can be characterized *via* a combination of facial AUs. Automatic AU detection has been a vital task for facial expression analysis, with a variety of applications in psychological and behavioral research, mental health assessment, and human-computer interaction (Bartlett et al., 2003; Zafar and Khan, 2014). Therefore, a reliable AU detection system is of vital importance for precise human emotion analysis.

Benefiting from the promising advancement in deep learning research, the performance and accuracy of AU detection has been improved by virtue of the convolutional neural network (CNN) based approaches in recent years (Li et al., 2017a,b, 2018a,b, 2020a; Corneanu et al., 2018; Jacob and Stenger, 2021). However, the CNN-model-based AU detection approaches are quite data starved. What is worse is that AU annotation is time-consuming, labor-intensive, cumbersome, and error-prone. Thus, many existing works propose to exploit the auxiliary information for precise AU detection, e.g., Yang et al. (2021) proposed to use the semantic embedding and visual feature (SEV-Net) for AU detection. SEV-Net obtains AU semantic embeddings through both intra-AU and inter-AU attention components to capture the relationships among words within each sentence that describes individual AU. Li and Shan (2021) use the categorical facial expression images as auxiliary training data to boost the AU detection performance in a meta-learning manner. These pioneering works have inspired us to use a large amount of unlabeled facial videos to learn the AU representation unsupervised, as the unlabeled facial videos are easy to obtain and they consist of a large amount of subjects with diverse facial expressions.

Recently, self-supervised learning (SSL) has shown promising potential in learning discriminative features from the unlabeled data *via* various different manually defined pretext tasks (Wang et al., 2020; Cai et al., 2021; Hu et al., 2021; Kotar et al., 2021; Luo et al., 2021; Sun et al., 2021). For the task of AU detection, Li et al. (2019b) proposed to predict the optical flow caused by AUs and poses between two randomly sampled facial frames in a video sequence. The optical flow of the AUs and poses are then linearly combined to obtain the overall displacements between the two sampled faces. Lu et al. (2020) leveraged the temporal consistency to learn the AU feature *via* a self-supervised temporal ranking constraint. To capture the AU correlations in an input facial image, Yan et al. (2021) disentangled the global feature into multiple AU-specific features *via* a contrastive loss and then compute the feature for each AU by aggregating the features from the other AU-specific features with a transformer component. To bridge the performance gap between the fully supervised and self-supervised AU detection methods, we propose a self-supervised pseudo signal based on the temporally predictive coding (TPC) to capture the temporal characteristics of the AUs. Specially, we construct a model that combines an AU feature extraction network with a convolutional gated recurrent unit (GRU) unit (Zonoozi et al., 2018), and a prediction head on top of the GRU that can make temporal predictions. We train the constructed model *via* TPC loss, which will be detailed in Section 3.1.

To further learn the per-frame discriminativeness between the sibling facial frames within a video clip, we propose a frame-wisely temporal contrastive learning mechanism. The AU detection model is tasked to perceive the temporal consistency and frame-wisely discriminativeness self-supervised. The AU detection backbone is trained end-to-end with the linear combination of the two contrastive losses on the unlabeled facial videos. Afterward, we additionally train a linear classifier with the pre-trained AU detection backbone with the scarce AU annotations.

In summary, the core contributions of this work can be summarized as follows:

1. We introduce self-supervised TPC for facial AU representation learning. TPC does not rely on AU annotations to learn the discriminative AU representations.
2. To further enhance the discriminability of the AU representation, TPC consists of a frame-wisely temporal contrastive learning constraint. TPC is capable of perceiving the temporal consistency and frame-wisely discriminativeness self-supervised.
3. Experimental results demonstrate the advantages of the proposed TPC over other state-of-the-art self-supervised AU detection methods on two popular AU datasets. Image retrieval results show that the learned AU representation in TPC is superior in spotting and capturing the AU similarities between different faces.

## 2. Related Work

A number of AU detection approaches have been proposed recently (Zhao et al., 2016; Li et al., 2017a,b; Li and Shan, 2021). AU detection approaches are deep learning-based mostly. Since AU actually means the movement of the facial muscles, many approaches detect the active/inactive states of AUs locally (Zhao et al., 2016; Li et al., 2017a,b). Among them, Zhao et al. (2016) used a locally connected convolutional layer to learn the AU-specific convolutional filters. SEV-Net (Yang et al., 2021) exploited the AU semantic word embedding as the auxiliary labels. FAUT was (Jacob and Stenger, 2021) proposed to capture the relationships between AUs *via* a transformer. These supervised AU detection methods need manually labeled training facial data. As training images are scarce, these methods often overfit on a specific dataset and cannot generalize well.

Recently, self-supervised (Wiles et al., 2018; Li et al., 2019b, 2020b; Lu et al., 2020) and weakly-supervised (Peng and Wang, 2018; Zhao et al., 2018) methods have been proposed to learn the deep learning-based models from unlabeled or partially labeled images. The former usually adopts the manually defined pseudo supervisory signals to learn the facial AU representation (Li et al., 2019b, 2020b; Lu et al., 2020). Among them, Fab-Net (Wiles et al., 2018) was trained to map a source facial frame to a target facial frame *via* estimating an optical flow field between the source and the target faces. Twin-cycle autoencoder (TCAE and TAE) (Li et al., 2019b, 2020b) were proposed to learn the pose-invariant facial action features by estimating the respective optical flows for the poses and AUs *via* the cycle-consistency in the image and representations. Lu et al. (2020) proposed a temporally sensitive triplet-based metric learning to learn the facial AU representations *via* capturing the temporal AU consistency. It actually learns to rank the neighboring faces from the sequential frames in the correct order. Our proposed TPC differs from previous methods in three aspects. First, TPC is self-supervised in the pre-training stage. Second, TPC does not crop the regional AU features to learn the region-specific AU feature. Instead, it uses an abundant number of unlabeled videos to enhance the AU detection performance. Finally, TPC is proposed to encode the temporal dynamics and consistencies to encode the characteristics of the facial AUs.

## 3. Method

**Figure 1:**
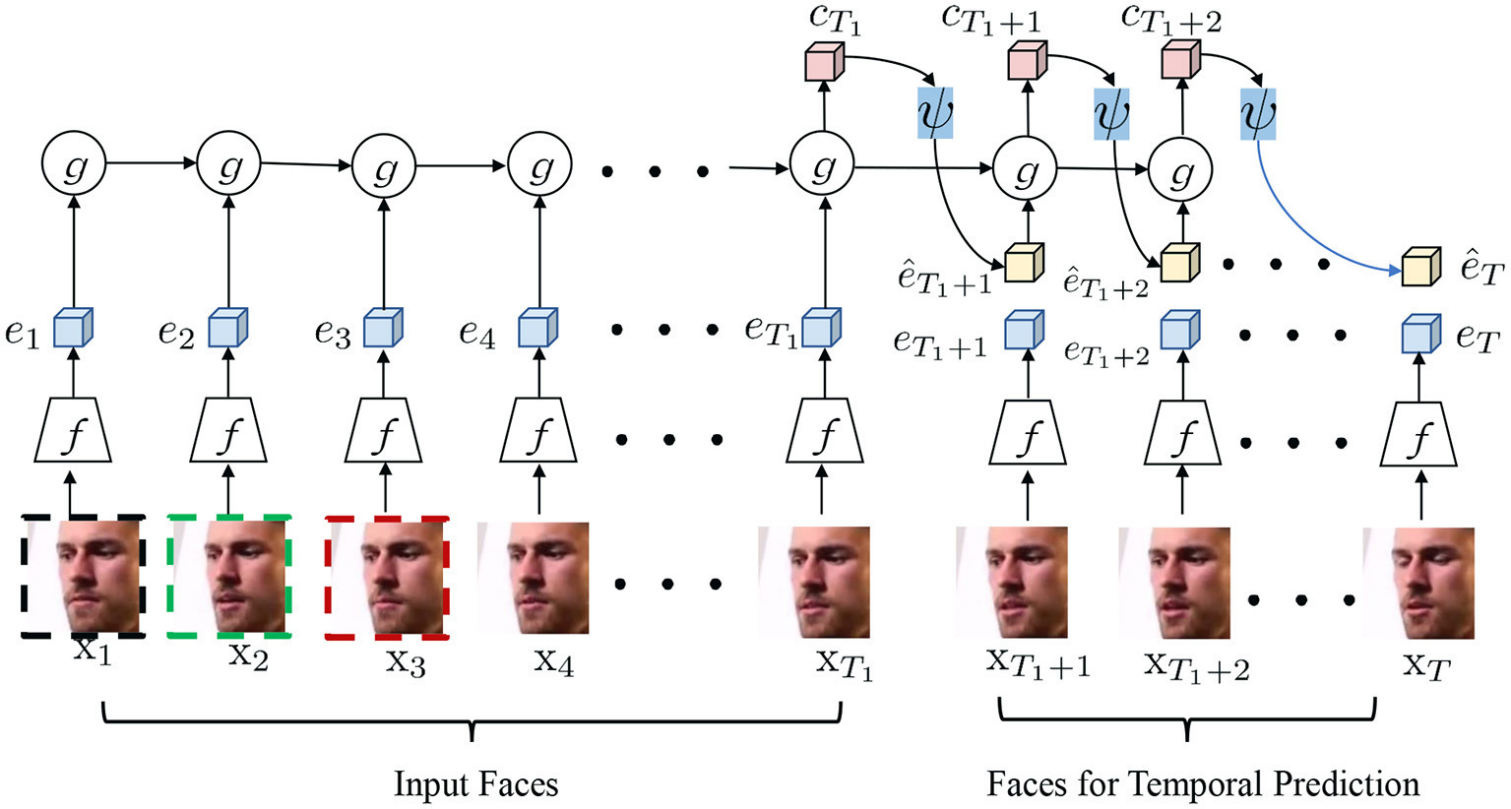
Main idea of the proposed self-supervised temporally predictive coding (TPC) for facial AU representation learning. Given a facial sequence with *T* faces, we use the preceding *T*_1_ faces as input and exploit the left faces for temporal prediction. Besides, we randomly sampled some triplets in each facial sequence to perceive the temporal consistency and frame-wisely discriminativeness self-supervised. ψ takes the context representation *c*_*t*_ as input and estimates the features for the future frame recursively. Better viewed in color and zoom in.

Figure 1 illustrates the main framework of the proposed TPC for AU representation learning. Given an input facial sequence sampled from an unlabeled facial video, TPC first extracts the convolutional feature maps of each face *via* a commonly-used backbone network such as ResNet-50. Second, TPC learns the discriminativeness between different facial frames *via* temporal contrastive learning. We will introduce the proposed TPC and present the temporal contrastive learning paradigm in our proposed TPC as below.

### 3.1. Temporal Predictive Coding

Videos are very appealing as a data source for self-supervision as there are many forms of pseudo signal. In detail, the self-supervision in the video sequence generally originates from three types: spatial, spatio-temporal, and sequential. Among the three kinds of self-supervised signal, spatial supervision can be derived from the structures in the static frame, spatio-temporal supervision naturally reflects the correlation across the different frames, and sequential supervision signifies the temporal coherence. Therefore, we exploit the sequential self-supervision to learn a robust model for facial AU detection that is capable of capturing the temporal dynamics as well as temporal consistency of the facial AUs.

**Figure 2:**
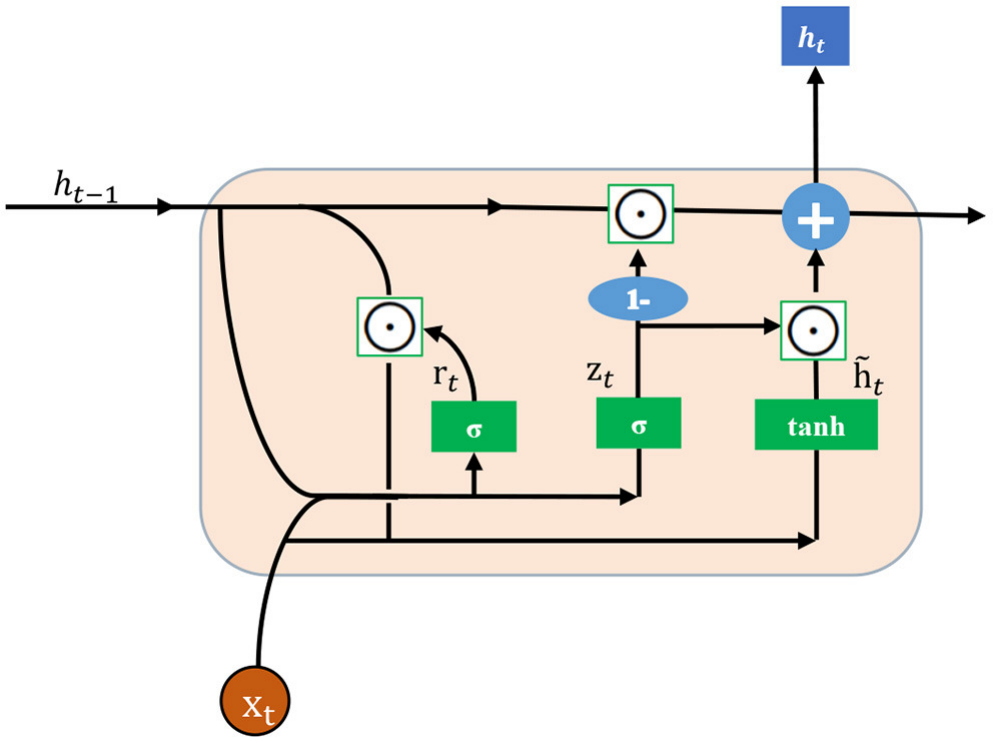
Illustration of the convolutional gated recurrent unit (GRU).

Let $\text{X}={\left\{x_{t}\right\}}_{t=1}^{T}$ denotes a consecutive sequence of *T* facial frames within an unlabeled video, where ${\text{x}}_{t}\in {\mathbb{R}}^{H\times W\times C}$ means the input *t*-th facial image of size *H*×*W*×*C*. Our goal here is to learn a model that predicts a slowly varying semantic representation based on the recent past. As illustrated in Figure 1, we partition a facial video clip into two parts: input part I and output part O:


(1) $$\begin{array}{r} \text{I}={\left\{{\text{x}}_{t}\right\}}_{t=1}^{T_{1}}, \end{array}$$

(2) $$\begin{array}{r} \text{O}={\left\{{\text{x}}_{t}\right\}}_{t=T_{1}+1}^{T}, \end{array}$$

where *T*_1_ is the length of the input facial sequence. First, a backbone network *f*(.) maps each facial frame x_*t*_ to its latent convolutional map representation $e_{t}\in {\mathbb{R}}^{H^{\prime }\times W^{\prime }\times C^{\prime }}$, organized as height × width × channels. Then, we use a convolutional GRU to aggregate the sequential latent representations into a context representation *c*_*t*_. Mathematically, GRU uses the same gated principal of LSTM but with a simpler architecture. The below equations describe the mathematical model for the GRU:


(3) $$\begin{array}{r} z_{t}=\sigma \left(W_{hz}h_{t-1}+W_{xz}e_{t}+b_{z}\right), \end{array}$$

(4) $$\begin{array}{r} r_{t}=\sigma \left(W_{hr}h_{t-1}+W_{xr}e_{t}+b_{r}\right), \end{array}$$

(5) $$\begin{array}{r} {ĥ}_{t}=\Phi \left(W_{h}\left(r_{t}⊙h_{t-1}\right)+W_{x}e_{t}+b\right), \end{array}$$

(6) $$\begin{array}{r} c_{t}=h_{t}=\left(1-z_{t}\right)⊙h_{t-1}+z⊙{ĥ}_{t}, \end{array}$$

where *h*_*t*_ is the hidden state, *r*_*t*_ and *z*_*t*_ are the reset gate value and update gate value at frame *t*. The functions σ(.) and Φ(.) denote the sigmoid and tangent activation functions, respectively. The reset gate *r*_*t*_ can decide whether or not to forget the previous activation. ⊙ means the element-wise multiplication. Figure 2 shows the main idea of the convolutional GRU.

With the encoded context representation *c*_*t*_, we exploit a prediction head ψ to predict the convolutional latent representation of the feature. In detail, ψ takes the context representation *c*_*t*_ as input and estimates the features for the future frame recursively:


(7) $$\begin{array}{r} e_{t+1}=\psi \left(c_{t}\right), \end{array}$$

(8) $$\begin{array}{r} e_{t+2}=\psi \left(c_{t+1}\right), \end{array}$$

where *c*_*t*_ means the context feature from time step 1 to *t*, and *e*_*t*+1_ means the estimated latent convolutional feature of the time step *t*+1. Similarly, we can predict the latent convolutional feature maps for the *t*+2 facial frame, in a recursive manner. Such a recursive TPC manner enforces the prediction to be conditioned on all previous predictions and observations. The intuition behind the TPC is that the model is tasked to infer future AU semantics from the context representations *c*_*t*_ and thus *c*_*t*_ has to encode temporal consistency and dynamics of the facial AUs.

The learning of the TPC is accomplished *via* a noise contrastive estimation, where our goal is the classify the real from the noisy samples. We denote the feature vector in each spatial location of the encoded and the predicted convolutional feature maps as *e*_*i,k*_ and ê_*i,k*_, where *i* denotes the temporal index and *k* means the spatial index in the convolutional features, *k*∈{(1, 1), (1, 2), ⋯ , (*H*′, *W*′)}. Finally, we can formulate the learning objective as follows:


(9) $$\begin{array}{r} L_{pred}=-\underset{i,k}{\sum }\text{log}\frac{\text{exp}\left({ê}_{i,k}\cdot e_{i,k}\right)}{\sum_{j,m}\text{exp}\left({ê}_{i,k}\cdot e_{i,m}\right)}. \end{array}$$

The goal of $L_{pred}$ is to classify the positive pair (ê_*i,k*_, *e*_*i,k*_) among a set of constructed pairs. A positive pair consists of two elements that are located in the same spatial location and at the same time step. All the other pairs (ê_*i,k*_, *e*_*j,m*_) that satisfy (*i, k*)≠(*j, m*) are negative pairs. $L_{pred}$ is optimized such that the similarities of the positive pairs are higher than the similarities of the negative pairs. While the proposed TPC can spot the temporal consistency and dynamics of the input facial sequences, the discriminativeness of the nearby facial frames can be further enhanced so that the encoded AU representation can be more discriminative. We will explain how we use the temporal contrastive learning paradigm to achieve this goal in the next section.

### 3.2. Temporal Contrastive Learning

To learn the frame wisely discriminativeness of the input facial images, we introduce a temporal contrastive learning goal by adding multiple triplet losses (Schroff et al., 2015), each measuring the pairwise distance between the adjacent frames to the anchor frame. Learning to rank through triplet loss actually trains an AU detection backbone that learns to make the distance between the anchor and the positive face smaller than the distance between the anchor and the negative face.

Let us denote a triplet that consists of three facial frames as (*x*_*a*_, *x*_*p*_, *x*_*n*_), where *x*_*a*_, *x*_*p*_, and *x*_*n*_ mean the anchor face, positive sample, negative sample, respectively. Note that *x*_*a*_, *x*_*p*_, and *x*_*n*_ are consecutive facial frames randomly sampled from the input facial sequence $\text{X}={\left\{x_{t}\right\}}_{t=1}^{T}$. Intuitively, (*x*_*a*_, *x*_*p*_) should have more similar facial expressions than (*x*_*a*_, *x*_*n*_) because the time interval is smaller between *x*_*a*_ and *x*_*p*_. Inspired by intuition, we randomly sampled *M* triplets from the input facial sequence X and expect that the sum of *M* triplet losses would enable the AU detection backbone to learn to perceive the facial expression difference in the nearby facial frames. The learning target of the proposed temporal contrastive learning paradigm can be formulated as:


(10) $$\begin{array}{r} L_{tcl}={\left[D\left(f\left(x_{a}^{i,1}\right),f\left(x_{p}^{i,j}\right)\right)-D\left(f\left(x_{a}^{i,1}\right),f\left(x_{n}^{i,j+1}\right)\right)+m\right]}_{+}, \end{array}$$

where *D* is the cosine similarity of the input frame pairs. *i* is the sequence index, *j* is the frame index within the *i*-th input facial sequence. *m* is the margin that ensures $L_{tcl}$ will not be zero until the difference between the distances of the negative and positive frame from the anchor is greater than *m*. For each training facial sequence with *T* faces, we randomly sampled *P* triplets.

### 3.3. Overall Training Objective of TPC

For pre-train, we use the linear combination of $L_{pred}$ and $L_{tcl}$ as below:


(11) $$\begin{array}{r} L_{total}=L_{pred}+\lambda L_{tcl}, \end{array}$$

where λ means the importance of the temporal triplet loss, which will be discussed in the experimental section.

For AU detection, we finetune the pre-trained model with the annotated AU labels. Mathematically, we exploit the multi-label sigmoid cross-entropy loss for optimizing the AU classification head and the pre-trained backbone model, which can be formulated as:


(12) $$\begin{array}{r} L^{AU}=-\overset{M}{\underset{m}{\sum }}z^{m}\log{ẑ}^{m}+\left(1-z^{m}\right)\log\left(1-{ẑ}^{m}\right), \end{array}$$

where *M* denotes the number of facial AUs. *z*^*m*^ denotes the *m*-th ground truth AU annotation of the input AU sample. ẑ^*m*^ means the predicted AU score. *z*_*i*_∈{0, 1} means the labels w.r.t the *i*th AU. 0 means the AU is inactive, and 1 means the AU is active.

## 4. Experiment

### 4.1. Implementation Details

We adopted ResNet-18 (He et al., 2016) as the backbone network for pretrain. We optimized the proposed backbone model *via* a batch-based stochastic gradient descent method. During training, we set the batch size as 64 on 4 GPU units and the initial learning rate as 0.001. For each video, we randomly sampled *T* = 10 consecutive faces for training, we used the first 8 eight faces as the input and the left 2 faces for prediction. Additionally, we randomly sampled *P* = 4 triplets from each facial sequence for temporal contrastive learning. During finetuning, we dropped the convolutional GRU and added a linear classifier layer for AU prediction. We set the momentum as 0.9 and the weight decay as 0.0005. We use the popular Voxceleb dataset (Nagrani et al., 2020) for pre-training. The dataset consists of about 6,000 subjects and hundreds of thousands of videos. All the videos only contain a subject with varying expressions and no AU or facial expression annotations.

#### 4.1.1. Datasets and Evaluation Metric

For AU detection, we adopted the denver intensity of spontaneous facial action (DISFA) (Mavadati et al., 2013) and binghamton-pittsburgh 3D dynamic spontaneous facial expression database (BP4D) (Zhang et al., 2013) datasets. BP4D consists of a total of 328 videos recorded for 41 subjects (18 men and 23 women). A total of 8 different experimental tasks are evaluated on the 41 subjects, and their spontaneous facial AUs variations were recorded in the videos. There are nearly 14,0000 frames with 12 facial AUs labeled. DISFA contains 27 participants. Each participant is asked to watch a video to elicit his/her facial expressions. The facial AUs are annotated with intensities ranging from 0 to 5. There are about 130,000 AU-annotated images in the DISFA dataset by setting the images with intensities greater than 1 as active. For the two datasets, the facial images are split into 3-fold in a subject-independent manner. We used the 3-fold cross-validation and adopted 12 AUs in BP4D and 8 AUs in DISFA dataset for evaluation.

We adopted F1-score to evaluate the performance of the proposed AU detection method. The F1-score can be calculated as $F1=\frac{2RP}{R+P}$, where *R* and *P*, respectively, denote the recall and precision. We also use the average F1-score over all the evolved AUs (Ave) to evaluate the overall facial AU detection precision.

### 4.2. Experimental Results

For the supervised methods, we compare the proposed TPC with deep region and multi-label (DRML) (Zhao et al., 2016), enhancing and cropping net (EAC-Net) (Li et al., 2017b), deep structure inference network (DSIN) (Corneanu et al., 2018), local relationship learning with person-specific shape regularization (LP-Net) (Niu et al., 2019), semantic relationship embedded representation learning (SRERL) (Li et al., 2019a), uncertain graph neural networks (UGN) (Song et al., 2021), semantic embedding and visual feature net (SEV-Net) (Yang et al., 2021) and facial action unit detection with transformers (FAUT) (Jacob and Stenger, 2021), meta auxiliary learning (MAL) (Li and Shan, 2021). It is worth noting that some of the AU detection approaches (Li et al., 2017b, 2019a; Corneanu et al., 2018; Jacob and Stenger, 2021) learn the AU-specific representations with exclusive CNN branches *via* cropping the local facial regions. SEV-Net (Yang et al., 2021) proposes to learn robust visual features for AU detection via introducing the auxiliary AU descriptions. UGN (Song et al., 2021) learn to model the uncertainty of the AU annotations.

For the self-supervised methods, we compare the proposed TPC with TCAE (Li et al., 2019b), TAE (Li et al., 2020b), triplet ranking loss (TRL) (Lu et al., 2020). Among the compared methods, in TRL (Lu et al., 2020) proposed an aggregate ranking loss by taking the sum of multiple triplet losses to allow pairwise comparisons between the adjacent facial frames. In TRL, they learn to rank the faces through triplet loss involves training an encoder that learns to force the distance between the anchor face and the positive face smaller than the distance between the anchor face and the negative face.

Table 1 shows the AU detection accuracy comparison of our TPC and previous methods on BP4D dataset. TPC obtains comparable AU detection accuracy in the average accuracy. In detail, TPC shows its superiority over DRML, EAC-Net, DSIN, LP-Net, with +12.8%, +5.2%, +2.2%, +0.1% improvements, respectively. Notably, TPC does not rely on facial landmarks to extract specified local facial regions, which will bring out a heavy computation burden in the training and inference phase. Besides, TPC does not need to use auxiliary AU description word embeddings or a large amount of annotated facial expression data for auxiliary learning. As different AUs are associated with specific facial muscles and corresponds to fine-grained local facial regions, learning region-specific AU representations is beneficial. The success of the region-based AU detection approaches (Li et al., 2017b, 2019a, 2020b; Corneanu et al., 2018; Jacob and Stenger, 2021) have verified the benefits of the region-based AU detection approaches. We will explore this in future work.

**Table 1:** Action unit (AU) detection accuracy of the proposed temporally predictive coding (TPC) and state-of-the-art approaches on BP4D dataset.

**Methods**	**AU1**	**AU2**	**AU4**	**AU6**	**AU7**	**AU10**	**AU12**	**AU14**	**AU15**	**AU17**	**AU23**	**AU24**	**Ave**
DRML Zhao et al. (2016)	36.4	41.8	43.0	55.0	67.0	66.3	65.8	54.1	33.2	48.0	31.7	30.0	48.3
EAC-Net Li et al. (2017b)	39.0	35.2	48.6	76.1	72.9	81.9	86.2	58.8	37.5	59.1	35.9	35.8	55.9
DSIN Corneanu et al. (2018)	51.7	40.4	56.0	76.1	73.5	79.9	85.4	62.7	37.3	62.9	38.8	41.6	58.9
LP-Net Niu et al. (2019)	43.4	38.0	54.2	77.1	76.7	83.8	87.2	63.3	45.3	60.5	48.1	54.2	61.0
UGN Song et al. (2021)	54.2	46.4	56.8	76.2	76.7	82.4	86.1	64.7	51.2	63.1	**48.5**	53.6	63.3
SRERL Li et al. (2019a)	46.9	45.3	55.6	77.1	78.4	83.5	87.6	63.9	52.2	**63.9**	47.1	53.3	62.9
FAUT Jacob and Stenger (2021)	51.7	49.3	**61.0**	77.8	**79.5**	82.9	86.3	**67.6**	51.9	63.0	43.7	**56.3**	**64.2**
SEV-Net Yang et al. (2021)	**58.2**	**50.4**	58.3	**81.9**	73.9	**87.8**	87.5	61.6	**52.6**	62.2	44.6	47.6	63.9
MAL Li and Shan (2021)	47.9	49.5	52.1	77.6	77.8	82.8	**88.3**	66.4	49.7	59.7	45.2	48.5	62.2
TCAE Li et al. (2019b)	43.1	32.2	44.4	**75.1**	70.5	80.8	85.5	61.8	34.7	58.5	37.2	**48.7**	56.1
TAE Li et al. (2020b)	**47.0**	**45.9**	50.9	74.7	**72.0**	**82.4**	85.6	62.3	48.1	**62.3**	45.9	46.3	60.3
TRL Lu et al. (2020)	42.3	24.3	44.1	71.8	67.8	77.6	83.3	61.2	31.6	51.6	29.8	38.6	52.0
**TPC (Ours)**	43.2	44.6	**52.8**	72.6	71.9	84.9	**86.9**	**64.8**	**50.3**	61.5	**55.6**	43.7	**61.1**

*The best results in the supervised and self-supervised methods are illustrated in Bold*.

Table 2 shows the AU detection accuracy comparison of our TPC and previous methods on the DISFA dataset. TPC achieves slightly superior AU detection accuracy with the best state-of-the-art self-supervised AU detection methods in the average F1 score, with 0.8% improvements over TAE, 7.3% improvements over TCAE, and 12.9% improvements over TRL. Notably, TPC shows its superiority in AU1 (Inner Brow Raiser), AU2 (Outer Brow Raiser), AU6 (Cheek Raiser), AU12 (Lip Corner Puller), and obtains comparable AU detection performance in AU9 (Nose Wrinkler) and AU25 (Lips part). In summary, the benefits of the proposed TPC over other self-supervised AU detection methods can be summarized in 2-fold. First, TPC explicitly learns to encode the temporal evolution and consistency of the facial Aus in the temporal sequences. The self-attention mechanism in the transformer modules is capable of perceiving the local to global interactions between different facial AUs. Second, TPC incorporates the frame-wisely temporal contrastive learning into the self-supervised paradigm to further learn the per-frame discriminative-ness between the nearby facial frames. Thus, TPC is capable of perceiving the temporal consistency and the frame-wisely discriminativeness of the facial AUs self-supervised. The consistent improvements over other self-supervised AU detection methods have verified the feasibility of TPC. We will carry out an ablation study to investigate the contribution of the two components in TPC in the next section.

**Table 2:** Action unit detection accuracy of the proposed TPC and state-of-the-art approaches on the DISFA dataset.

**Methods**	**AU1**	**AU2**	**AU4**	**AU6**	**AU9**	**AU12**	**AU25**	**AU26**	**Ave**
DRML Zhao et al. (2016)	17.3	17.7	37.4	29.0	10.7	37.7	38.5	20.1	26.7
EAC-Net Li et al. (2017b)	41.5	26.4	66.4	50.7	**80.5**	**89.3**	88.9	15.6	48.5
OFS-CNN Han et al. (2018)	43.7	40.0	67.2	59.0	49.7	75.8	72.4	54.8	51.4
DSIN Corneanu et al. (2018)	42.4	39.0	**68.4**	28.6	46.8	70.8	90.4	42.2	53.6
SRERL Li et al. (2019a)	45.7	47.8	59.6	47.1	45.6	73.5	84.3	43.6	55.9
LP-Net Niu et al. (2019)	29.9	24.7	72.7	46.8	49.6	72.9	93.8	65.0	56.9
FAUT Jacob and Stenger (2021)	46.1	48.6	72.8	**56.7**	50.0	72.1	90.8	55.4	**61.5**
SEV-Net Yang et al. (2021)	**55.3**	53.1	61.5	53.6	38.2	71.6	95.7	41.5	58.8
UGN Song et al. (2021)	43.3	48.1	63.4	49.5	48.2	72.9	90.8	59.0	60.0
MAL Li and Shan (2021)	**43.8**	**39.3**	**68.9**	47.4	**48.6**	**72.7**	**90.6**	52.6	**58.0**
TCAE Li et al. (2019b)	15.1	15.2	50.5	48.7	23.3	72.1	82.1	52.9	45.0
TAE Li et al. (2020b)	21.4	19.6	**64.5**	46.8	**44.0**	73.2	**85.1**	**55.3**	**51.5**
TRL Lu et al. (2020)	18.7	27.4	35.1	33.6	20.7	67.5	68.0	43.8	39.4
**TPC (Ours)**	**22.8**	**30.8**	59.6	**53.9**	42.7	**75.3**	82.1	51.6	52.3

*The best results in the supervised and self-supervised methods are illustrated in Bold*.

#### 4.2.1. Ablation Study

Table 3 shows the ablation experimental results. In Table 3, we show the accuracy variations with a different self-supervised components, and show the influence with different λ. As shown in Table 3, TPC shows the best AU detection performance with the linear combination of $L_{pred}$ and $L_{tcl}$ with λ = 0.1. It means both components in TPC contribute to its success in learing discriminative AU representations. Without either of the two self-supervised targets, TPC will show degraded AU detection accuracies. Besides, TPC also suffers from low accuracy with λ = 1.0 and λ = 10.0, which suggests the two self-supervised learning targets should be appropriately balanced to achieve the discriminative AU representations.

**Table 3:** Ablation studies on the BP4D and DISFA datasets.

**Methods**	**BP4D**	**DISFA**
$L_{pred}$	58.7	49.8
$L_{tcl}$	57.9	50.8
λ = 10.0	55.2	47.1
λ = 1.0	59.3	48.6
λ = 0.1	61.1	52.3

## 5. Conclusion

Within this paper, we aim to propose a self-supervised pseudo signal based on TPC to capture the temporal characteristics of the facial AUs in the sequential facial frames. To further learn the per-frame discriminativeness between the nearby faces, TPC incorporates the frame-wisely temporal contrastive learning into the self-supervised paradigm. The proposed TPC can be pre-trained without AU annotations, which facilitates making use of a large amount of unlabeled facial videos to learn the AU features that are robust to other undesired nuisances. Compared with supervised facial AU detection methods, TPC obtains comparable AU detection performance. Besides, TPC is superior to other self-supervised AU detection approaches. For future work, we will explore learning to perceive the regional and structural AU features in the temporal contrastive learning paradigm.

## Data Availability Statement

The original contributions presented in the study are included in the article/supplementary material, further inquiries can be directed to the corresponding author.

## Author Contributions

CW completed the algorithm design and wrote all parts of the manuscript. CW and ZW cooperatively conducted the experimental evaluation and cooperatively gave a detailed experimental analysis. ZW carefully checked the manuscript and polished the paper. Both authors have carefully read, polished, and approved the final manuscript.

## Conflict of Interest

The authors declare that the research was conducted in the absence of any commercial or financial relationships that could be construed as a potential conflict of interest.

## Publisher's Note

All claims expressed in this article are solely those of the authors and do not necessarily represent those of their affiliated organizations, or those of the publisher, the editors and the reviewers. Any product that may be evaluated in this article, or claim that may be made by its manufacturer, is not guaranteed or endorsed by the publisher.
